# Down syndrome as risk factor for respiratory syncytial virus hospitalization: A prospective multicenter epidemiological study

**DOI:** 10.1111/irv.12431

**Published:** 2016-12-30

**Authors:** Manuel Sánchez‐Luna, Constancio Medrano, Julián Lirio, the RISK‐21 Study Group, José Antonio Hurtado Suazo, Manuela Peña, Eduardo Narbona López, José Uberos Fernández, Enrique Blanca Jover, Antonio Bonillo Perales, Javier Díes Delgado, Mª Ángeles Ortega Montes, Jesús de la Cruz Moreno, Joaquín Ortiz Tardío, Mª Victoria Esteban, Leticia Millán, Carlos Salido Peracaula, Gloria María Quesada Trujillo, José David Martínez Pajares, Simón Pedro Lubián López, Salvador Ariza Aranda, Mª Purificación Ventura Faci, Sofía Valle Guillén, Olga Bueno Lozano, Segundo Rite Gracia, Miguel Ángel García Cabezas, Natalia Bejarano Ramírez, Mª Carmen Fresneda Machado, Andrés Martínez Gutiérrez, Sara Rellán Rodríguez, Marianela Marcos Temprano, Elena Ortega Vicente, Fernando Centeno Malfaz, Laura San Feliciano Martín, Ana Remesal Escalero, Francisca Benito Zaballos, Ricardo Closa‐Monasterolo, Silvia Franch Salvadó, Israel Fernando Anquela Sanz, Joaquim Bosch Castells, Alberto Trujillo Fagundo, Emma Ametller Malfaz, Mario José Sánchez Fernández, Eduard Solé Mir, Wilfredo Coroleu Lletget, Ignacio Arroyo Carrera, María Taboada Perianes, Mª Luz Couce Pico, María José Fernández Seara, Mª Yolanda Ruiz del Prado, Mª Luisa Poch Olive, Elena Maderuelo, Susana Zeballos, Ana Leal Orozco, Cristina Ruíz Serrano, Félix Omeñaca, Esperanza Escribano Palomino, Luis García Guereta, Vicente Bosch Jiménez, José Diego Gutiérrez Sánchez, Manuel Cidrás Pidre, Natividad Viguria Sánchez, Concepción Goñi Orayen, Ana Aguirre Unceta‐Barrenechea, Alberto Pérez Legorburu, Mª José Palao Ortuño, Maribel Giner Crespo, Ángel González Muñoz, Mª Isabel Izquierdo Macián, Rafael Gómez Zafra, Juan Mayordomo Colunga, Bárbara Montes Zapico, María Rosón Varas, María Mora Matilla, Ignacio Oulego Herroz, Mª Teresa Prada Pereira, Miguel Ángel Arias Consuegra, Laura Castells Vilella, Jesús Antonio Mairal Cazcarra, Silvia Yévenes Ruiz, Consorci Sanitàri de Terrassa, Isabel Sáez Díez, Mercedes García Reymundo, Esther Piñán López, Javier Fernández Sarabia, Sofía Hernández Cáceres, Mª Gloria López Lois, Cristina Olivas, Mª José Rivero Martin, Susana de las Heras Ibarra, Mercedes Cuadrado, Jose Tomás Ramos Amador, Sara Guillén Martin, Alfonso Cañete Díaz, Julia Sopeña Corvino, Francisco Javier González‐Valcárcel Sánchez –Puelles, Jose Cambra Sirera, Begoña Pérez García, Maria Isabel Jiménez Candel, Elisa Cueto Calvo, Leonor Guardia, Julián Lirio Casero, Gemma Ginovart Galiana, Esther López Bernal, Lorenzo Sánchez de León, Maria Jesus Ferrandez, Jose Luis Quiles, H. de Elche, Maria Rimblas, Susana Larrosa Capaces, H. San Joan de Reus

**Affiliations:** ^1^ Neonatology DivisionHospital Materno InfantilHospital General Universitario Gregorio MarañónUniversidad ComplutenseMadridSpain; ^2^ Cardiology DivisionHospital Materno InfantilHospital General Universitario Gregorio MarañónUniversidad ComplutenseMadridSpain; ^3^ Social Pediatric DivisionHospital Infantil Universitario Niño JesúsMadridSpain

**Keywords:** Down syndrome, hospitalization, palivizumab, prospective birth cohort study, respiratory syncytial virus

## Abstract

**Background:**

Respiratory syncytial virus (RSV) infection in childhood, particularly in premature infants, is associated with significant morbidity and mortality.

**Objectives:**

To compare the hospitalization rates due to RSV infection and severity of disease between infants with and without Down syndrome (DS) born at term and without other associated risk factors for severe RSV infection.

**Patients/Methods:**

In a prospective multicentre epidemiological study, 93 infants were included in the DS cohort and 68 matched by sex and data of birth (±1 week) and were followed up to 1 year of age and during a complete RSV season.

**Results:**

The hospitalization rate for all acute respiratory infection was significantly higher in the DS cohort than in the non‐DS cohort (44.1% vs 7.7%, *P*<.0001). Hospitalizations due to RSV were significantly more frequent in the DH cohort than in the non‐DS cohort (9.7% vs 1.5%, *P*=.03). RSV prophylaxis was recorded in 33 (35.5%) infants with DS. The rate of hospitalization according to presence or absence of RSV immunoprophylaxis was 3.0% vs 15%, respectively.

**Conclusions:**

Infants with DS showed a higher rate of hospitalization due to acute lower respiratory tract infection and RSV infection compared to non‐DS infants. Including DS infants in recommendations for immunoprophylaxis of RSV disease should be considered.

## 1 Introduction

Respiratory syncytial virus (RSV) infection in childhood, particularly in premature infants, is associated with significant morbidity, hospitalization rates including neonatal intensive care unit admission, healthcare burden and mortality.^1^, ^2^, ^3^, ^4^ Infants in their first year of age are the most vulnerable population and globally 40% of RSV infections progress to lower respiratory tract infections (LRTI).^5^, ^6^ Most hospitalized children are younger than 6 months of age. Decreased lung function at birth, prematurity, chronic lung disease, age <6 weeks, and congenital heart disease (CHD) has been reported as classic risk factors for severe disease after RSV infection.^7^, ^8^, ^9^

Down syndrome (DS) is the most common chromosomal abnormality among live‐born infants. DS is characterized by a variety of dysmorphic features and congenital malformation, including CHD. In addition, respiratory infections are still the most important cause of mortality in DS at all ages.^10^, ^11^, ^12^ Also, DS has been recently recognized as a risk factor for RSV LRTI. In a birth cohort study of 219 children with DS and 276 siblings of the cohort used as controls, a higher incidence of hospitalizations due to RSV LRTI in the DS group was observed, independently of the presence or absence of CHD as compared to controls (9.9% vs 0.7%, respectively).^13^ Using statewide hospitalization data for children with DS for 1995 through 2006 from the Colorado Health and Hospital Association database, children with DS had a significantly higher risk than did those without DS for being hospitalized for RSV LRTI (odds ratio [OR] 5.99, 95% confidence interval [CI] (5.38‐6.68), even in the absence of coexisting underlying conditions (OR 3.5, 95% CI: 3.10‐4.12).^14^ In a comparison of hospitalization rates for acute respiratory tract infection in children younger than 24 months between those with significant CHD without DS and children with DS (with or without CHD), the hospital admission rate was 19.1% in the DS group and 11% in the non‐DS group (OR 1.9, 95% CI: 1.3‐2.7).^15^ Moreover, significant differences were found in the incidence of hospital admissions due to RSV between children with and without DS (7.8% vs 3.2%, respectively, OR 2.6, 95% CI: 1.4‐4.7).^15^

The present prospective multicenter epidemiological study was conducted to compare the hospitalization rates due to RSV‐related acute respiratory infections between infants with DS born at term and without other associated risk factors (CHD or chronic pulmonary disease) and infants without DS and no risk factors for RSV infection.

## 2 Patients and Methods

This was a prospective multicenter epidemiological study carried out in the Services of Neonatology and/or Paediatric Cardiology of 50 acute‐care hospitals throughout Spain. The primary objective of the study was to determine whether neonates with DS and without associated risk factors had a higher risk of hospitalization due to RSV infection compared with neonates without DS, matched by age and birth date. The study was conducted in infants up to 1 year of age over the RSV season. The secondary objective was to assess disease severity in the two cohorts.

Between 1 September 2012 and 1 September 2013, neonates born at term who fulfilled the inclusion criteria were recruited for the study, and those included were followed up to the first complete RVS season (from October to March 2012‐2013 or 2013‐2014). Written informed consent from the parents or legal guardians was obtained prior to inclusion in the study. The study protocol was approved by the Ethics Committee of the participating hospitals. The study was conducted in accordance with the Declaration of Helsinki principles (2008 and subsequent revisions) and Good Clinical Practice Guidelines.

Male and female infants with DS born at term and/or followed at the participating hospitals, aged less than 1 year at the beginning of the RSV season (September 30, 2012 or September 30, 2013), were eligible. The diagnosis of DS was established clinically and confirmed by genetic testing. The cohort of controls included infants matched by sex and date of birth (±1 week). Exclusion criteria for all participants were as follows: presence of hemodynamically significant CHD documented by echocardiography and a clinical cardiological evaluation performed by paediatric cardiologist, bronchopulmonary dysplasia defined as the need of supplemental oxygen for ≥28 days after birth, premature infants with gestational age <35 weeks, nosocomial RSV infection defined as symptoms of LRTI and RSV antigen beginning >72 hours after admission to the hospital for any reason, parents or legal guardians not being fluent in Spanish, major neonatal surgery and refusal to sign the informed consent.

Infants in both cohorts were visited at the time of inclusion in the study (RSV hospitalization), and at the end of the epidemic season, data recorded during the first visit and hospital admissions due to RSV infection that occurred during the epidemic season were checked. Follow‐up telephone calls were also performed. At baseline, sociodemographic and clinical data were recorded. In relation to the primary objective of the study, the number of admissions to the hospital due to RVS infection during the epidemic season was registered. Hospitalization was defined as a hospital stay of more than 24 hours, the reason of which was an acute respiratory infection characterized by an episode of bronchiolitis, pneumonia, nasopharyngitis or other respiratory tract diseases, and attributable to RSV according to the viral diagnosis. The diagnosis of RSV was established at least by a rapid antigen detection test but without excluding the use of other methods, such as cell cultures or molecular techniques.

In relation to the secondary objective of the study, the following variables were collected: length of hospital stay (days), interventions/medications administered during in‐hospital care, admission to the paediatric intensive care unit (PICU) and length of stay in the PICU, duration of assisted ventilation, duration of oxygen therapy, presence of pulmonary hypertension and death.

Risk or protective factors for RSV hospitalization were also recorded, including sex, weight, age of the parents, number of siblings less than 11 years of age, day care attendance and early care, number of siblings attending nursery or school, twins, active smokers among family members, mother smoking during pregnancy, number of members in the household, ethnicity, education level of parents/legal guardians, urban or rural place of residence, previous hospitalizations for other reasons, weeks of gestation at birth, breastfeeding duration, prophylaxis for RSV before each hospitalization (number of doses), vaccines received (compliance with the vaccination calendar) and admission to the hospital due to an acute respiratory infection (diagnosis on discharge and causative pathogens). Risk factors in the DS cohort, such as thyroid disease, epilepsy, autoimmune diseases, intestinal obstruction and gastroesophageal reflux disease, were also recorded.

### 2.1 Statistical analysis

Assuming a prevalence of hospitalization due to RSV infection in the population of children of ≤2 years of age of 2.4%,^14^ a sample size of 253 children for each study cohort was estimated to detect a clinically relevant effect,^14^ with a probability associated with the difference of 0.055. This would result in a rate of hospitalizations of 7.9% in the cohort with DS. To account for a 10% loss rate, 279 infants in each cohort were planned.

Qualitative data are expressed as frequencies and percentages, and quantitative data as mean and standard deviation (SD) or median and interquartile range (IQR) (25th‐75th percentile). Categorical variables were compared with the chi‐square test or the Fisher's exact test, and continuous variables with the Wilcoxon rank‐sum test or the Mann‐Whitney *U*‐test. Univariate and multivariate logistic regression analysis was performed to assess the association between baseline variables and hospitalization due to RSV adjusted by group. Odds ratio (OR) and 95% confidence intervals (CI) were calculated.

## 3 Results

A total of 167 infants were included in the study, 97 in the DS cohort and 70 in the non‐DS cohort. However, four infants (two in each cohort) were lost to follow‐up, the diagnosis of DS was not confirmed in one, and the selection criteria were not met in one. Therefore, 161 evaluable patients (63.3% males) with a mean (SD) age of 7.8 (5.7) months were included in the analysis. There were 93 infants in the DS cohort and 68 in the non‐DS cohort.

Baseline characteristics of the two cohorts are shown in Table 1. A higher incidence in the DS group compared to the non‐DS group included lower birthweight and gestational age at birth, older ages of fathers and mothers, lower education levels of the parents, and higher percentages of nursery attendance or early care, siblings attending nursery or school, and number of people in household. The percentage of infants previously hospitalized because of RSV infection or due to other reasons, as well as the percentage of infants that had received prophylaxis against RSV, was also significantly higher in the DS group. Concomitant diseases and use of concomitant medications were also significantly more frequent in the DS cohort than in the non‐DS cohort (Table 2).

**Table 1:** Sociodemographic and clinical variables at baseline

Variables	All infants(n=161)	DS cohort(n=93)	Non‐DS cohort(n=68)	*P* value
Sex, no. (%)				
Males	102 (63.3)	59 (63.4)	43 (63.2)	.978
Females	59 (36.6)	34 (36.6)	25 (36.8)	
Age, days, mean (SD)	235.4 (172.0)	223.2 (162.1)	252.1 (184.6)	.425
Weight, kg, mean (SD)	6.5 (2.6)	6.0 (2.4)	7.2 (2.7)	.007
Ethnicity, no. (%)				
Caucasian	141 (87.6)	78 (81.7)	65 (95.6)	.058
Other^a^	20 (12.4)	17 (18.3)	3 (4.4)	
Gestational age at birth, weeks, mean (SD)	38.3 (1.5)	37.7 (1.4)	39.1 (1.3)	<.001
Breastfeeding duration, months, mean (SD)	94.2 (110.6)	82.6 (106.7)	109.5 (114.5)	.069
Twins, no.(%)	3 (1.9)	3 (3.2)	0	.263
Father's age, years, mean (SD)	36.3 (6.1)	37.2 (7.0)	35.1 (4.4)	.007
Mother's age, mean (SD)	35.0 (5.1)	35.8 (5.7)	33.8 (3.9)	.004
Siblings <11 y of age, median (IQR)	0 (0‐1)	1 (0‐1)	0 (0‐1)	.109
Nursery attendance or early care, no. (%)	63 (39.1)	47 (50.5)	16 (23.5)	.0004
Siblings in nursery/school, median (IQR)	0.5 (0‐1)	1 (0‐1)	0 (0‐1)	.007
Place of residence, no. (%)				
Urban	125 (77.6)	66 (71.0)	59 (86.8)	.017
Rural	36 (22.4)	27 (29.0)	9 (13.2)	
Current smokers, no. (%)				
Father	43 (75.4)	25 (71.4)	18 (81.8)	.336
Mother	24 (42.1)	16 (45.7)	8 (36.4)	.486
Family members	57 (35.4)	35 (37.6)	22 (32.5)	.457
Members in the household, median (IQR)	4 (3‐4)	4 (3‐5)	3 (3‐4)	.0006
Education level of the parents, no. (%)				
Low	16 (9.9)	14 (15.1)	2. (2.9)	.013
Medium	57 (35.4)	35 (37.6)	22 (32.5)	
High	87 (54.0)	43 (46.2)	44 (64.7)	
Previous hospitalizations, no. (%)				
RSV infection	11 (6.8)	10 (10.7)	1 (1.4)	.025
Other reasons	40 (24.8)	35 (37.6)	5 (7.3)	<.001
Prophylaxis against RVS, no. (%)	33 (20.5)	33 (35.5)	0	<.001
Doses, mean (SD)	3.8 (1.6)	3.8 (1.6)	0	
Compliance vaccination calendar, no. (%)	157 (97.5)	89 (95.7)	68 (100)	.138

a Black, Hispanic, Asiatic, Arabs.

**Table 2:** Concomitant diseases and concurrent medication at baseline

Variables	All infants(n=161)	DS cohort(n=93)	Non‐DS cohort(n=68)
Any concomitant disease	66 (50.0)	55 (59.1)^a^	11 (16.2)
Non‐hemodynamically significant CHD	26 (16.1)	26 (27.9)	0
Thyroid disease	10 (6.2)	10 (10.7)	0
Epilepsy	2 (1.2)	2 (2.1)	0
Gastroesophageal reflux	5 (3.1)	2 (2.1)	3 (4.4)
Other	40 (24.8)	32 (34.4)	8 (11.8)
Concurrent medication	32 (19.9)	29 (31.2)^a^	3 (4.4)
Thyroid hormone	10 (4.7)	10 (7.1)	0
Anticonvulsants	6 (2.8)	6 (4.3)	0
Bronchodilators	24 (11.2)	19 (13.6)	5 (6.8)
Inhaled corticosteroids	13 (6.1)	13 (9.3)	0
Oral corticosteroids	7 (3.3)	7 (5.0)	0
Other	12 (5.6)	10 (7.1)	2 (2.7)

a *P*<.0001 for the comparison of the DS and non‐DS cohorts.

During the study period, a total of 192 hospitalizations were recorded (127 in the DS cohort and 65 in the non‐DS cohort). A total of 61 hospitalizations (31.7%) were due to acute respiratory tract infections. The rate of hospitalization for acute respiratory infection was significantly higher in the DS cohort than in the non‐DS cohort (44.1% [56/127] vs 7.7% [5/65], *P*<.0001). As shown in Table 3, a single pathogen was identified in 15 infants (RSV 11, pneumococcus 1, influenza 1 and other pathogens 2) and polymicrobial infection in 2. RSV was identified as the causative pathogen in 11 cases of which 10 (two episodes in nine infants) occurred in the DS cohort and one in the non‐DS cohort. Also, hospitalizations due to RSV were significantly more frequent in the DH cohort than in the non‐DS cohort (9.7% vs 1.5%, *P*=.03).

**Table 3:** Acute respiratory infections, hospitalization‐related data and prophylaxis for RSV

Variables	All infants(n=61)	DS cohort(n=56)	Non‐DS cohort(n=5)
Diagnoses			
Acute respiratory tract infection/influenza	13 (21.3)	13 (23.2)	0
Pneumonia	13 (21.3)	12 (21.4)	1 (20.0)
Bronchiolitis	33 (54.1)	28 (50.0)	5 (100)
Other	23 (37.7)	23 (41.1)	0
Causative pathogens			
Single pathogen	15 (24.6)	14 (25.0)	1 (20.0)
Pneumococcus	1 (6.7)	1 (7.1)	0
Influenza	1 (6.7)	1 (7.1)	0
RSV	11 (73.3)	10 (71.4)	1 (100)
Other	2 (13.3)	2 (14.3)	0
Polymicrobial infection	1 (1.6)	1 (1.8)	0
None	5 (8.2)	5 (8.9)	0
Prophylaxis for RSV	13 (23.2)	13 (21.3)	0
Doses before hospitalization			
1	2 (15.4)	2 (15.4)	0
2	2 (15.4)	2 (15.4)	0
3	1 (7.7)	1 (7.7)	0
4	1 (7.7)	1 (7.7)	0
5	6 (46.1)	6 (46.1)	0
6	1 (7.7)	1 (7.7)	0

**Figure 1:**
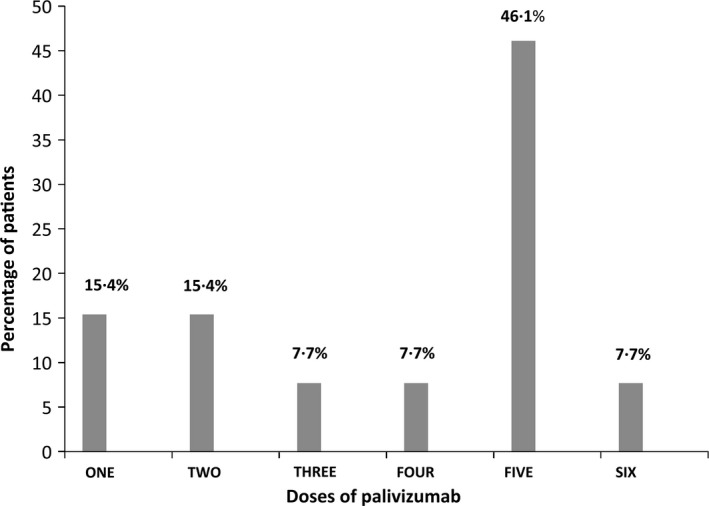
Distribution of patients with Down syndrome according to the number of doses of palivizumab received before hospitalization

The percentage of breastfeed infants at the time of hospital admission was similar in the two cohorts (26.4% vs 20%). Bronchiolitis, upper respiratory tract infection/influenza and pneumonia were the most common clinical diagnoses. Among patients admitted to the hospital due to acute respiratory infection, prophylaxis for RSV with palivizumab was recorded in 23.2% of cases in the DS cohort and in none in the non‐DS cohort, with 46% of hospitalized patients having received five doses of the drug (Table 3). The distribution of DS patients according to the doses of palivizumab is shown in Figure 1.

Rapid antigen‐based testing for RSV detection was performed in 30 cases in the DS cohort and in one in the non‐DS cohort, being positive in 10 (33.3%) and 1 (100%) cases, respectively. In the DS cohort, other techniques included cell culture (21 cases, 1 positive), RSV nucleic acids (two cases, one positive), microarrays (one case, one negative) and DNA polymerase chain reaction (one case, one negative). In the DS cohort of 93 infants, RSV prophylaxis was recorded in 33 (35.5%) infants. In the remaining 60 infants, prophylaxis was not administered. The rate of hospitalization for any acute respiratory tract infection episode according to presence or absence of RSV prophylaxis was 3.0% (1/33) vs 15% (9/60). However, prophylaxis was recorded in only one of the nine SD infants admitted to the hospital on 10 occasions (two episodes in one infant) with the diagnosis of RSV. In this infant, the length of hospitalization was 5 days and paediatric ICU admission was not necessary. The mean length of hospitalization in the whole cohort of DS admitted to the hospital because of acute respiratory infections was 7.3 (9.1) days.

Differences in baseline variables between the groups with and without RSV prophylaxis were not observed, except for a higher percentage of infants with non‐significant hemodynamic CHD in the group with prophylaxis (48.5% vs 16.7%, *P*=.001) and mean (SD) older ages of both fathers (39.3 [5.5] vs 36.0 [7.6], *P*=.01) and mothers (38.3 [4.3] vs 34.5 [5.9], *P*=.002).

In the univariate logistic regression analyses adjusted by DS and non‐DS groups, the only independent predictors of hospitalization due to RSV infection were number of siblings less than 11 years of age (OR=4.85, 95% CI: 1.49‐15.75, *P*=.008) and previous hospitalizations for any cause (OR=15.24, 95% CI: 2.85‐81,35, *P*=.001). Other baseline variables including age at the time of recruitment, sex, father's and mother's ages, nursery attendance or early care, siblings in nursery/school, current smokers among family members, number of people in the household, education level of the parents, place of residence, gestational age at birth, duration of breastfeeding and prophylaxis against RSV were not significant. In the multivariate analysis, only previous hospitalization was an independent risk factor for hospital admission due to RSV (OR=20.11, 95% CI: 3.53‐114.32, *P*=.0007).

In relation to the disease severity recorded in the hospitalizations due to acute respiratory tract infection between the groups of 56 DS infants and five non‐DS infants, differences in length of hospital stay, paediatric ICU admission, length of ICU stay, need of assisted ventilation, supplemental oxygen therapy and pulmonary hypertension were not found (Table 4). No deaths were registered.

**Table 4:** Variables related to severity of illness in hospitalized patients due to an acute respiratory infection

Variables	DS cohort(n=56)	Non‐DS cohort(n=5)	*P* value
Length of hospital stay, days, mean (SD)	7.3 (9.1)	3.0 (2.1)	0.295
Paediatric ICU admission, no. (%)	8 (14.3)	0	1.000
Length of ICU stay, days, mean (SD)	7 (4.2)	0	
Assisted ventilation, no. (%)	2 (3.6)	0	1.000
Supplemental oxygen therapy, no. (%)	30 (53.6)	1 (20.0)	0.195
Pulmonary hypertension, no. (%)	1 (1.8)	0	1.000
Duration of pulmonary hypertension, days, median	51.0	0	

## 4 Discussion

The present study shows significant differences in the hospitalization rates due to acute respiratory tract infection in two cohorts of infants with and without DS, with a higher rate in the DS cohort. These cohorts were matched by age and date of birth, and no hemodynamically significant CHD was present. Also, the rate of hospitalization due to RSV infection during the epidemic season was significantly higher in the DS cohort than in infants without DS. Nursery attendance a known risk factor for LRTI was quite higher in the DS group making them more at risk. Other interesting findings included the fact that 33 of the 93 infants in the DS cohort had received prophylaxis for RSV, but prophylaxis was recorded in only one of the nine infants admitted to the hospital on 10 occasions (two episodes in one infant) with the diagnosis of RSV. The fact of whether this infant had an indication for prophylaxis that was not recorded is unknown. In this single infant with prophylaxis, the duration of hospitalization was shorter than in DS infants hospitalized because of acute respiratory tract infections. Although based on data of one patient only, the rate of hospitalization associated with RSV in infants with DS was 3.0% in those with RSV prophylaxis and 15% in those without prophylaxis.

The data from this study align with data reported by other authors^14^, ^15^, ^16^ and provide evidence of the contribution of DS to the risk of hospitalization with RSV. In a previous multicenter study (CIVIC study) of respiratory infections in children younger than 24 months with significant CHD carried out during two seasons (October to April from 2004 to 2006), the hospitalization rate for acute respiratory tract infection, the associated risk factors and compliance of preventive measures were evaluated.^15^ The rate of hospitalization for respiratory infection was 13.4%. Bronchiolitis was the most common clinical picture, which is consistent with our data. On the other hand, RSV was the most frequently identified infectious pathogen, which is also in agreement with our findings. In the CIVIC study, chromosomal abnormalities (22q11 deletion and trisomy 21), siblings less than 11 years old, prematurity and incomplete prophylaxis against RSV were risk factors for respiratory infections. In this study, however, cardiological factors were not identified as risk factors for respiratory infections because the presence of hemodynamically significant CHD was an exclusion criterion. A further epidemiological study (CIVIC 21 study)^16^ carried out in Spain in which hospitalization rates for acute respiratory tract infection in children younger than 24 months with significant CHD without DS were compared to those of children with DS with and without CHD, also revealed higher rates of acute respiratory tract infection in patients with DS (19.1% vs 11%). The specific admission rate due to RSV was 7.8% in the DS group and 3.2% in the non‐DS group. Immunoprophylaxis against RSV in DS patients was 39.9% vs 83.4% in infants without DS. This low rate of immunoprophylaxis in DS patients was also observed in our cohort. Differences between the present RISK‐21 study and the previous CIVIC 21^16^ study include the following: (i) study population (children younger than 24 months with significant CHD compared to children younger than 24 months with DS, with and without associated CHD in the CIVIC 21 study^16^; children up to 1 years of age with DS and children up to 1 year of age without DS matched by sex and date of birth [±1 week] in the current study); (ii) objectives (assessment of hospitalization rates due to respiratory infection^16^ vs hospitalization rates due to RSV infection); and (iii) recruitment period (from October 2006 to March 2007 in the CIVIC 21 study^16^, and neonates born at term between September 2012 and September 2013 in the present study).

Different studies have shown that infants with medical conditions such as DS are at higher risk of hospitalization due to RSV infection.^17^ Palivizumab has been recommended for high‐risk infants to prevent severe RSV‐associated lower respiratory tract illness. In a systematic review of observational studies focusing on the real‐world effectiveness of palivizumab, recommendations for the use of immunoprophylaxis with palivizumab to reduce RSV‐associated hospitalization rates were clearly supported in premature infants born at gestational age <33 weeks and in children with chronic lung and heart diseases.^18^ In other high‐risk children, including those with DS, cystic fibrosis and haematological malignancies, data are limited. However, based on findings of the present study, including DS infants in recommendations for immunoprophylaxis of RSV disease should be considered. Interestingly, a recent retrospective cohort database study in which treatment with palivizumab was an exclusion criterion, 842 children with DS and 632,358 children without DS were compared.^19^ The rate of RSV hospitalization was 9.6% for the group of DS children vs 2.8% for those without DS. Moreover, DS had a greater hazard ratio (HR 3.46, 95% CI: 2.75‐4.37) than other studied risk factors (CHD, prematurity, neuromuscular disease, cystic fibrosis, congenital airways anomalies) for RSV hospitalization. Patients with DS had a significantly higher risk ratio for requiring respiratory support. Finally, the risk of RVS hospitalization in patients with DS was evaluated in patients with DS up to 36 months of age. The sensitivity analysis shows that the increased risk for RSV hospitalization persists in DS in children who were 24‐36 months old.

The present findings should be interpreted taking into account some limitations of the study, which include the prospective observational design and the inclusions of a lower number of infants because of difficulties in the recruitment of patients in the DS cohort due to the decline of babies born with DS in Spain. Also, differences in the number of patients with DS (n=93) and controls (n=68) should be considered. The study design for the control group, sex and date of birth (±1 week) matching may account for the difference. Moreover, only one epidemic season could be finally evaluated, and the new statistical power actually is with the new numbers. Disparity in testing for RSV between the cohorts, probably reflecting the daily clinical practice, is also a limitation of the study.

In summary, the rates of hospitalization for acute respiratory tract infection as well as for RSV infection were significantly higher in the DS cohort as compared to non‐DS infants, but differences in severity of disease between the two cohorts were not observed. In the DS cohort, the rate of hospitalization showed a fivefold increase in the absence of previous RSV immunoprophylaxis. Based on this observation and given that infants with DS and without associated risk factors for RSV were selected for the study, including DS infants in recommendations for immunoprophylaxis of RSV disease should be considered.

## Conflict of Interest

M. Sánchez Luna and C. Medrano provide assistance as consultants for Abbvie Spain, S.L.U.J. Lirio has collaborated with Abbvie Spain, S.L.U., as an investigator in the RISK‐21 study.

## Financial Disclosure Statement

AbbVie provided funding for this study. AbbVie participated in the design, collection and analysis of data and reviewed and approved this manuscript. AbbVie has no commercial interests in the results of this article.

## Appendix 1

### Members of the RISK‐21 Study Group

José Antonio Hurtado Suazo and Manuela Peña, Hospital Virgen de las Nieves, Granada; Eduardo Narbona López, José Uberos Fernández and Enrique Blanca Jover, Hospital San Cecilio, Granada; Antonio Bonillo Perales, Javier Díes Delgado and María Ángeles Ortega Montes, Hospital Torrecárdenas, Almería; Jesús de la Cruz Moreno, Joaquín Ortiz Tardío, María Victoria Esteban, Leticia Millán, Carlos Salido Peracaula and Gloria María Quesada Trujillo, Hospital Materno‐Infantil, Centro Hospitalario de Jaén, Jaén; José David Martínez Pajares, Hospital Comarcal de Antequera, Málaga; Simón Pedro Lubián López, Hospital Puerta del Mar, Cádiz; Salvador Ariza Aranda, Hospital Universitario Carlos Haya, Málaga; María Purificación Ventura Faci, Sofía Valle Guillén and Olga Bueno Lozano, Hospital Clinico Universitario Lozano Blesa, Zaragoza; Segundo Rite Gracia, Hospital Universitario Miguel Servet, Zaragoza; Miguel Ángel García Cabezas, Natalia Bejarano Ramírez and María Carmen Fresneda Machado, Hospital General Universitario de Ciudad Real, Ciudad Real; Andrés Martínez Gutiérrez, Hospital General de Albacete, Albacete; Sara Rellán Rodríguez, Marianela Marcos Temprano and Elena Ortega Vicente, Hospital Clínico Universitario de Valladolid, Valladolid; Fernando Centeno Malfaz, Hospital Universitario Río Hortega, Valladolid; Laura San Feliciano Martín, Ana Remesal Escalero and Francisca Benito Zaballos, Hospital Universitario de Salamanca, Salamanca; Ricardo Closa‐Monasterolo and Silvia Franch Salvadó, Hospital Joan XXIII, Tarragona; Israel Fernando Anquela Sanz and Joaquim Bosch Castells, Hospital General de Granollers, Granollers, Barcelona; Alberto Trujillo Fagundo, “Emma Ametller Malfaz and Mario José Sánchez Fernández, Hospital Universitari Dr. Josep Trueta, Girona; Eduard Solé Mir, Hospital Uiversitari Arnau de Vilanova, Lleida; Wilfredo Coroleu Lletget, Hospital Universitai Germans Trias i Pujol, Badalona, Barcelona; Ignacio Arroyo Carrera, H. San Pedro de Alcántara, Cáceres; María Taboada Perianes, Complexo Hospitalario Universitario A Coruña, A Coruña; María Luz Couce Pico and María José Fernández Seara, Hospital Clínico Universitario de Santiago de Compostela, Santiago de Compostela; María Yolanda Ruiz del Prado and María Luisa Poch Olive, Hospital de San Pedro‐Rioja Salud, Logroño; Manuel Sánchez Luna, Elena Maderuelo and Susana Zeballos, Hospital General Universitario Gregorio Marañón, Madrid; Ana Leal Orozco and Cristina Ruíz Serrano, Fundación Jiménez Díaz, Madrid; Félix Omeñaca, Esperanza Escribano Palomino and Luis García Guereta, Hospital Infantil La Paz, Madrid; Vicente Bosch Jiménez, José Diego Gutiérrez Sánchez and Manuel Cidrás Pidre, Hospital Universitario Virgen de la Arrixaca, Murcia; Natividad Viguria Sánchez and Concepción Goñi Orayen, Hospital Virgen del Camino, Pamplona; Ana Aguirre Unceta‐Barrenechea and Alberto Pérez Legorburu, Hospital de Basurto, Bilbao; María José Palao Ortuño and Maribel Giner Crespo, Hospital de Manises, Valencia; Ángel González Muñoz, Hospital de La Ribera, Alzira, Valencia; María Isabel Izquierdo Macián, Hospital Universitari i Politècnic La Fe de Valencia, Valencia; Rafael Gómez Zafra, Hospital General de Valencia, Valencia; Juan Mayordomo Colunga and Bárbara Montes Zapico, Hospial San Agustín, Avilés; María Rosón Varas, María Mora Matilla and Ignacio Oulego Herroz, Hospital de León, León; María Teresa Prada Pereira and Miguel Ángel Arias Consuegra, Hospital del Bierzo, León; Laura Castells Vilella, Hospital General de Catalunya, Sant Cugat del Vallés, Barcelona; Jesús Antonio Mairal Cazcarra and Silvia Yévenes Ruiz, Consorci Sanitàri de Terrassa, Terrassa, Barcelona; Isabel Sáez Díez, Mercedes García Reymundo and Esther Piñán López, Hospital de Mérida, Badajoz; Javier Fernández Sarabia and Sofía Hernández Cáceres, Hospital Universitario de Canarias, Santa Cruz de Tenerife; María Gloria López Lois and Cristina Olivas, Hospital Universitario Príncipe de Asturias, Madrid; María José Rivero Martin, Susana de las Heras Ibarra and Mercedes Cuadrado, Hospital de Fuenlabrada, Madrid; Jose Tomás Ramos Amador and Sara Guillén Martin, Hospital de Getafe, Madrid; Alfonso Cañete Díaz, Hospital Infanta Sofía, San Sebastián de los Reyes, Madrid; Julia Sopeña Corvino, Francisco Javier González‐Valcárcel Sánchez –Puelles, Hospital Infanta Elena, Madrid; Jose Cambra Sirera, Begoña Pérez García and Maria Isabel Jiménez Candel, Hospital Luis Alcanyis, Xátiva, Valencia; Elisa Cueto Calvo and Leonor Guardia, Hospital Virgen de la Luz, Cuenca; Julián Lirio Casero, Hospital Niño Jesús, Madrid; Gemma Ginovart Galiana and Esther López Bernal, Hospital de la Santa Creu i Sant Pau, Barcelona; and Lorenzo Sánchez de León, Hospital de Móstoles, Madrid, Spain. Maria Jesus Ferrandez and Jose Luis Quiles, H. de Elche, Alicante; Maria Rimblas and Susana Larrosa Capaces, H. San Joan de Reus, Tarragona.
